# Metformin and Flutamide Combination Therapy’s Efficacy and Safety in Prostate Cancer Cell Lines

**DOI:** 10.1155/proc/9093252

**Published:** 2026-05-29

**Authors:** AhmadReza Rezaeian, Fatemeh Khatami, Seyed Reza Hosseini, Iman Menbari Oskouie, Akram Mirzaei, Rahil Mashhadi, Helia Azodian Ghajar, Mahdi Khoshchehreh, Ali Tavoosian, Seyed Mohammad Kazem Aghamir

**Affiliations:** ^1^ Urology Research Center, Tehran University of Medical Sciences, Tehran, Iran, tums.ac.ir; ^2^ School of Medicine, Tehran University of Medical Sciences, Tehran, Iran, tums.ac.ir; ^3^ Department of Pathology, University of California, Los Angeles, USA, berkeley.edu

**Keywords:** combination therapy, flutamide, metformin, prostate cancer

## Abstract

**Background:**

Considering the hepatotoxicity and other side effects associated with flutamide as a first‐line treatment for prostate cancer (PCa), this study aimed to evaluate metformin as a potential anticancer agent to reduce the required dose of flutamide and thereby minimize its adverse effects.

**Method:**

We assessed the influence of metformin, flutamide, and metformin–flutamide combination therapy on LNCaP, DU145, and PC3 cell lines, which represent human PCa. The tests include wound‐healing assay, colony formation assay (CFA), analysis of apoptosis (programmed cell death) and cell cycle by flow cytometry, gene expression at the mRNA level by real‐time PCR (*BAX/BCL2*, *E-cadherin*, *Snail*, *HIF1α*, *VEGFC*, and *KLK3* genes), and assessment of the treatments’ hepatotoxicity potential via measuring AST and ALT values.

**Result:**

To determine the IC50 (half‐maximal inhibitory concentration) values, cell lines were treated with different concentrations of the drugs. The IC50 values for metformin (800 μM) in the three cell lines and for flutamide (12 μM for PC3 and 10 μM for LNCaP/DU145), as determined by MTT assay, were confirmed by flow cytometry, indicating significant cell cycle arrest at the G0/G1 phase. The combination of metformin and flutamide significantly increased the *BAX/BCL2* mRNA ratio in all three cell lines (*p* < 0.0001) and downregulated the expression of *KLK3* (*p* < 0.01), *HIF1α* (*p* < 0.01), *VEGFC* (*p* < 0.001), and *EMT* pathway genes in PC3 and LNCaP (*p* < 0.01). Liver injury assessment reported a reduction in flutamide’s hepatotoxicity in combination with metformin.

**Conclusion:**

Metformin in combination with flutamide reduced its dose and increased the sensitivity of PCa cells to treatment. Additionally, it mitigated the hepatotoxic effects of flutamide. Therefore, this combination may represent a new treatment strategy for PCa.

## 1. Introduction

Prostate cancer (PCa) is the most common malignancy among men. According to GLOBOCAN 2023, PCa ranked as the second most commonly diagnosed cancer after lung cancer, with an incidence rate of 14.1% and 1,958,310 new cases worldwide [1]. In the early stages of PCa, androgen‐dependent lesions are typically observed, and the primary treatment involves androgen ablation. However, several challenges contribute to PCa being the second leading cause of cancer‐related death. The response to androgens is not permanent; over time, cancer cells may develop resistance and become androgen‐independent. In some cases, tumor recurrence occurs after treatment, and the cancer no longer responds to androgen therapies [2, 3]. Despite the availability of various combination therapies for managing androgen‐independent PCa, numerous clinical trials are still underway to identify newer and more effective treatment options. For over half a century, primary androgen deprivation therapy (ADT) has been known as the cornerstone of treatment [4].

Flutamide is the first nonsteroidal antiandrogen therapy with well‐established clinical use, making it a familiar intervention to healthcare providers. Based on clinical outcomes and research, side effects of flutamide include gynecomastia, breast sensitivity, hot flashes, diarrhea, and hepatotoxicity [5].

Metformin, a well‐known oral biguanide, is an inexpensive, safe, and popular drug [6]. Since the early 2000s, evidence has demonstrated the drug’s potential as an antineoplastic treatment [6, 7]. Metformin’s favorable outcomes in hormone‐sensitive cancers such as prostate and breast tumors have been repeatedly reported [8]. In addition to its anticancer properties, metformin may also help PCa patients in managing ADT‐related metabolic syndrome [9].

Notably, a cohort of over 87,000 patients demonstrated that veterans receiving concurrent metformin for diabetes mellitus and ADT for PCa had improved overall survival compared to those not receiving metformin [10].

Given the aforementioned advantages, this research aims to evaluate, for the first time, the molecular effects of combining flutamide and metformin in PCa cell lines.

### 1.1. Cell Culture

The LNCaP‐FGC‐10, DU145, and PC3 cell lines were sourced from the National Cell Bank of the Pasteur Institute with NCBI codes C439, C428, and C427, respectively. They were cultured in Gibco high‐glucose DMEM medium supplemented with 10% Gibco fetal bovine serum, 1000 units/mL Gibco penicillin, and 100 μg/mL Gibco streptomycin and maintained in a 37°C, 5% CO2 and humidified incubator. Flutamide (Iran Hormone Pharmaceutical Co.) and metformin (Abidi Pharma Co.) were prepared in appropriate solvents and added to the culture medium. Tests for mycoplasma were negative.

### 1.2. Cell Viability Assay

Three PCa cell lines were incubated in 96‐well plates (5 × 10^3 cells/well) and exposed to different concentrations of flutamide (ranging from 0 to 20 μM) and metformin (extending from 200 to 1200 μM). Then data were compared in 24‐, 48‐, and 72 h following interventions. The PCa cells’ viability was assessed via MTT assay. The optimal density [8] of the wells was read at a wavelength of 545 nm. Using Version 9 of GraphPad Prism software, dose–response diagrams were formed and IC50 values were determined.

### 1.3. Cell Lines’ Morphology

Morphological examination of PCa cell lines, LNCaP, DU145, and PC3, was performed to evaluate the effects of metformin and flutamide individually and in combination. First, 5 × 10^3 cells were seeded in each well of 96‐well plates. After 24 h, the desired drugs were added to the wells at the obtained concentrations. Then, 48 h after treatment, the medium on the cells was removed, then the cells were fixed with a 4% paraformaldehyde solution, and then washed twice with PBS, and finally, images were obtained with an inverted microscope (200x magnification).

### 1.4. Colony Formation Assay (CFA)

Cells (10^3 cells) were exposed to DMSO, metformin IC50, and flutamide IC50 over a 48 h period. Then cells were incubated for 14 days at 37°C in fresh media without any drug treatment to assess their colony formation ability. Subsequently, the cells were washed twice with PBS and stained with 0.2% w/v crystal violet solution for 30 min at 25°C. Employing an inverted microscope, colony formation (a colony considered to be a claustration of 50 or more cells) was evaluated in each group. To lessen the error rate, all tests were repeated independently on three separate occasions. ImageJ software (Version 1.53) was applied on images to count colony numbers.

### 1.5. Wound‐Healing Assay

Cells were seeded (5∗10^5 cells/well) and grown in a 6‐well plate to meet approximately 85% confluency. Afterward, a pipette tip was used to apply a vertical scratch in wells. Following PBS washing and an overnight serum starvation, each group was exposed to its mentioned treatment. At 24 h intervals, cell imaging was done, and cell migration capacity was calculated by measuring the space between the scratch edges. In order to quantify the rate, ImageJ software was employed to investigate all images.

### 1.6. Flow Cytometric Analysis of Apoptosis

Following the manufacturer’s guideline, an Annexin‐V and Propidium Iodide [11] kit (BioLegend; CAT Number: 640914) according to the manufacturer’s instructions was utilized to show the rate of viable, necrotic, and apoptotic cells. 1∗10^6 Cells were incubated at 37°C in DMEM with 10% FBS throughout the night. Then, the appropriate concentrations of drugs were added to each group and incubated for 48 h. On that occasion, cells were treated with Annexin‐V and PI and incubated at 37°C and darkness for a period of 15 min. The scatter plots were divided into four unique regions, each representing different cell populations. The annexin V^−^/PI^−^ (Alive Cells: Q4/Lower Left Quadrant), the annexin V^+^/PI^−^ (Early Apoptotic Cells: Q3/Lower Right Quadrant), the annexin V^+^/PI^+^ (Late Apoptotic: Q2/Upper Right Quadrant), and finally the annexin V^−^/PI^+^ (Necrotic Cells: Q1/Upper Left Quadrant). The measurement of apoptosis was conducted by assessing the proportion of annexin V^+^/PI^−^ cells through a flow cytometer. The obtained data were subsequently analyzed using FlowJo software (Tree Star Inc., Version 9.6.3, USA).

### 1.7. Flow Cytometric Analysis of DNA Cell Cycle

The cell cycle assessment was done through fixing LNCaP, DU145, and PC3 cells in 70% cold ethanol for a duration of 24 h. The 1∗10^6 cells were then washed twice with PBS and incubated with RNase I and 5∗10^2 μL PI at 37°C for 30 min. After staining, the cells were analyzed using a flow cytometer. FlowJo software (Tree Star Inc., Version 9.6.3) was recruited to process the results. Additionally, cell arrest in the sub‐G0/G1 phase was interpreted as an indicator of apoptosis.

### 1.8. Real‐Time PCR Analysis of Gene Expression

Initially, RNA extraction was performed using TriPure Isolation Reagent, followed by RNA concentration quantification by the Colibri Microvolume Spectrometer. Subsequently, cDNA synthesis was carried out using the Easy TM cDNA Synthesis Kit (Pars Toos Company). Real‐time PCR was performed on a QIAGEN thermocycler, with a 20 μL sample volume. The PCR reaction was validated through analyzing melting curves. B2M mRNA levels were set as the internal reference, and the 2^−ΔΔCT^ method was conducted to measure the relative expression values. The primers’ nucleotide sequences are demonstrated in Table 1. GraphPad Prism Version 10 software was employed to analyze the data.

**TABLE 1:** Nucleotide sequences of employed primers.

Gene	Accession number	Forward and reverse primers (5′–3′)	PCR product
*B2M*	TGTCTTTCAGCAAGGACTGGT	TGCTTACATGTCTCGATCCCAC	143
*Bcl2*	CTGCACCTGACGCCCTTCACC	CACATGACC CCACCGAACTCAAAGA	119
*Bax*	TGGAGCTGCAGAGGATGATTG	GAAGTTGCCGTCAGAAAACATG	95
*E-cadherin*	TCGTAACGACGTTGCACCAA	TTCGGAACCGCTTCCTTCAT	175
*Snail*	TAGCGAGTGGTTCTTCTGCG	AGGGCTGCTGGAAGGTAAAC	164
*HIF-1α*	GTGCCACATCATCACCATATAG	GCTTTCTCTGAGCATTCTGCAA	201
*VEGF-C*	GCTTCTTCTCTGTGGCGTGT	CTTTGCTTGCATAAGCCGTGG	150
*KLK3*	CGTGACGTGGATTGGTGCT	TTCCTGATGCAGTGGGCAGC	175

### 1.9. Liver Injury Indicators

Liver damage was assessed by examining the condition medium of treated 1∗10^6 cells using assay kits and enzyme‐linked immunosorbent assay (ELISA) methods. Quantification of aspartate transaminase (AST) and alanine transaminase [6] levels was conducted using ELISA kits sourced from My BioSource, Inc. (AST Cat No: MBS3801937 and ALT Cat No: MBS8801462). Following a 5‐min centrifuge at 3∗10^3 x g, the conditioned medium was obtained, and experiments were carried out carefully according to the manufacturer’s instructions. The standard curve was created through serial dilutions. All analysis was performed using a microplate reader outfitted with a 450 nm detection wavelength filter and 570 or 630 nm correction wavelength filters.

### 1.10. Statistical Analysis

The experiments were conducted in triplicate, with the findings expressed as the mean ± standard deviation (SD). For statistical evaluation, analysis of variance (ANOVA) and Student′s *t*‐test were employed. For comparing two combinations, a two‐way ANOVA was the appropriate method to assess significance. Finally, post hoc analysis (Tukey) was conducted for statistical corrections. Statistical significance, in comparison to the respective control group, was determined as follows: ^∗^ *p* < 0.05, ^∗∗^ *p* < 0.01, ^∗∗∗^ *p* < 0.001, and ^∗∗∗∗^ *p* < 0.001.

## 2. Result

### 2.1. Cell Viability Assay

Cytotoxic effects of metformin (0–1200 μM), flutamide (0–20 μM), and their combination were evaluated in LNCaP, DU 145, and PC3 cells following 24, 48, and 72 h of treatment. Half‐maximum inhibitory concentrations (IC50) of flutamide were 12 and 10 μM for PC3 and LNCaP/DU145 cells, respectively, and the metformin IC50 for all these three cell lines was found to be 800 μM in 48 h. Data showed drugs alone and in combination are cytotoxic in time‐ and dose‐dependent manners. The combination of two drugs significantly reduces the number of PCa cells (Figure 1). Due to the efficacy of metformin in combination with flutamide, we used an 800 μM dosage for metformin (Figure 1(a)) and 8 μM flutamide instead of 12 μM in PC3 and 10 μM in DU145 and LNCaP, respectively (Figure 1(b)).Finally, the combination dose of 800 μM metformin and 8 μM flutamide was chosen in 48 h (Figure 1(c)).

FIGURE 1MTT assay. Cell proliferation of prostate cancer cells (LNCaP, DU145, and PC3) following metformin (0–1200 μM), flutamide (0–20 μM), and their combination treatment (800–600 and 12–4 μM). The drug inhibited prostate cancer cells’ growth in time‐ and dose‐dependent manners. Determined half‐maximum inhibitory effects (IC50) are marked with stars.

**Figure:**
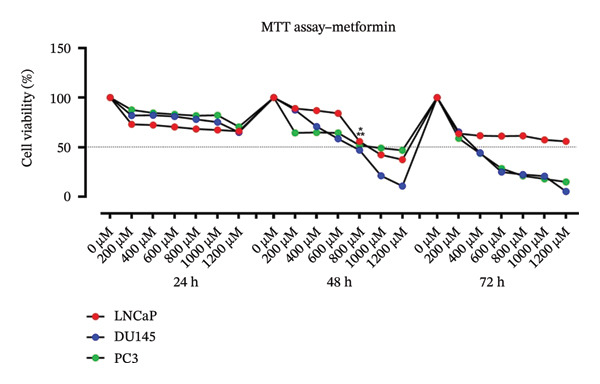
(a)

**Figure:**
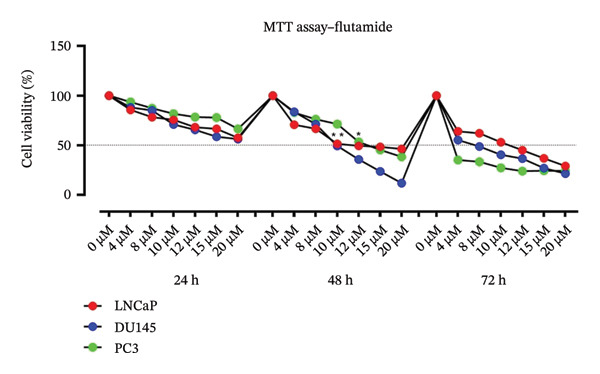
(b)

**Figure:**
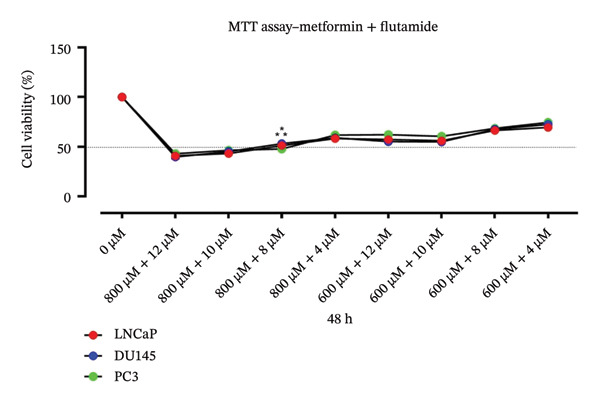
(c)

### 2.2. Evaluation of Cell Morphology

**FIGURE 2:**
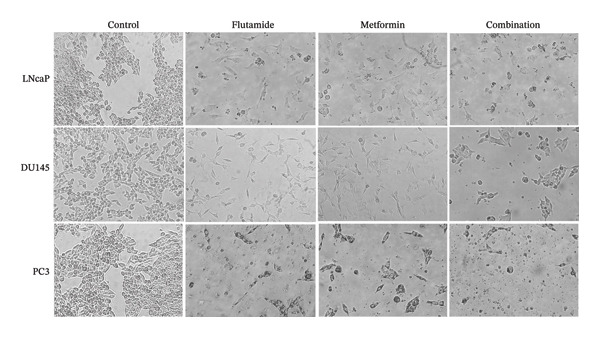
Cell morphology. Cell morphology of prostate cancer cells (LNCaP, DU145, and PC3). Following treatment, changes in cell morphologies were observed, including reduce cellular appendages and inhibited cell growth. Images were captured at 400x magnification.

Treatment of PCa cell lines with metformin, flutamide, or a combination of both resulted in a reduction in cell viability. In addition, surviving cells showed changes in morphology characterized by elongation, wrinkling, and shrinkage. An increase in cell debris was also observed. These morphological changes were most pronounced in the metformin–flutamide combination group compared to single‐agent treatment groups (Figure 2). After treatment, LNCaP and DU145 cell lines become more rounded, debris increases, and they lose their elongation, while the response of PC3 cells to single and combined treatment is such that the number of cells decreases, debris increases, and the cells become more elongated.

### 2.3. Evaluation of Colony Formation

**FIGURE 3:**
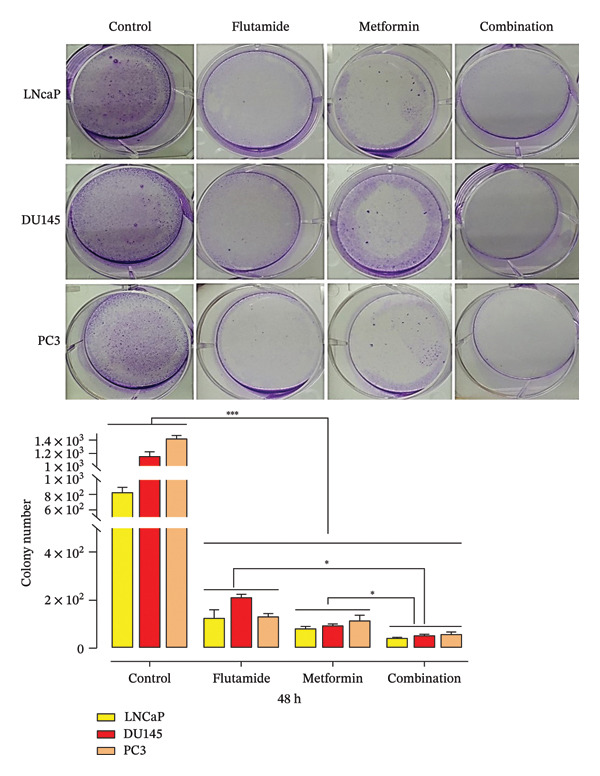
Colony formation assay. Colony‐forming potential of prostate cancer cell lines (LNCaP, DU145, and PC3). The combined treatment with metformin and flutamide significantly impaired the colony‐forming ability of cells compared to both the corresponding control and single‐agent treatment groups. Statistical significance was observed at ^∗^ *p* < 0.05, ^∗∗^ *p* < 0.01, ^∗∗∗^ *p* < 0.001, and ^∗∗∗∗^ *p* < 0.0001 relative to the control group.

Metformin and flutamide alone and in combination reduced the numbers of colonies compared with the untreated group. Thereby, exposing LNCaP, DU 145, and PC3 cells to metformin and flutamide IC50s hinders the cells’ ability to form colonies, with the most pronounced effect observed in metformin–flutamide groups across all cell lines. The results also demonstrated that metformin alone was more effective than flutamide in suppressing colony formation in LNCaP and DU145 cells. However, the combination treatment demonstrated the greatest reduction in colony formation in all three PCa cell lines (Figure 3).

### 2.4. Effects of Metformin and Flutamide on the Migration of the PCa Cells

**FIGURE 4:**
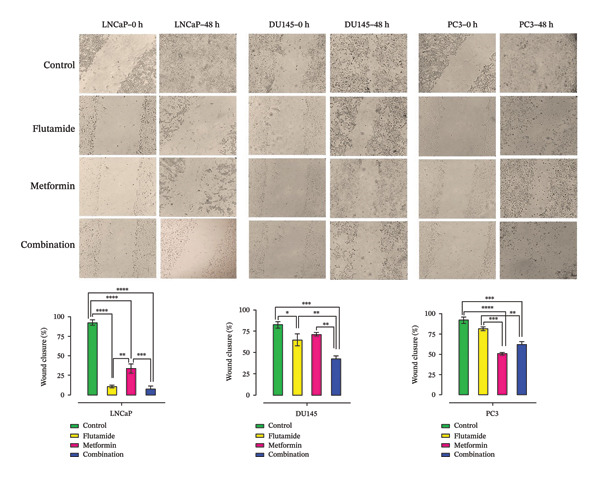
Migration assay. Cell migration ability of prostate cancer cells (LNCaP, DU145, and PC3). Following 48 h, treated groups demonstrated inhibition of migration abilities compared to the corresponding control groups. Statistical significance was distinct at *p* < 0.05, ^∗∗^ *p* < 0.01, ^∗∗∗^ *p* < 0.001, and ^∗∗∗∗^ *p* < 0.0001 compared to the control group.

Comparison of the gap widths between scratch edges demonstrated that all three treatments inhibit PCa cells’ migration ability after 48 h. The metformin–flutamide combination was most effective in reducing migration in DU145 and PC3 cells. However, in LNCaP cells, flutamide alone showed the strongest inhibitory effect. In contrast, the scratch area in untreated groups was significantly filled after 48 h. As shown in Figure 4, cells in the control group completely filled the gap after 48 h, while after treatment with metformin and flutamide, filling of the imaginary scratch occurred less. Flutamide prevented cell migration and scratch filling more than metformin, and this was more evident in cell line LNCaP than in DU145 and PC3 cells.

### 2.5. Metformin and Flutamide‐Induced Cell Cycle Arrest at the G0/G1 Phase in PCa Cells

**FIGURE 5:**
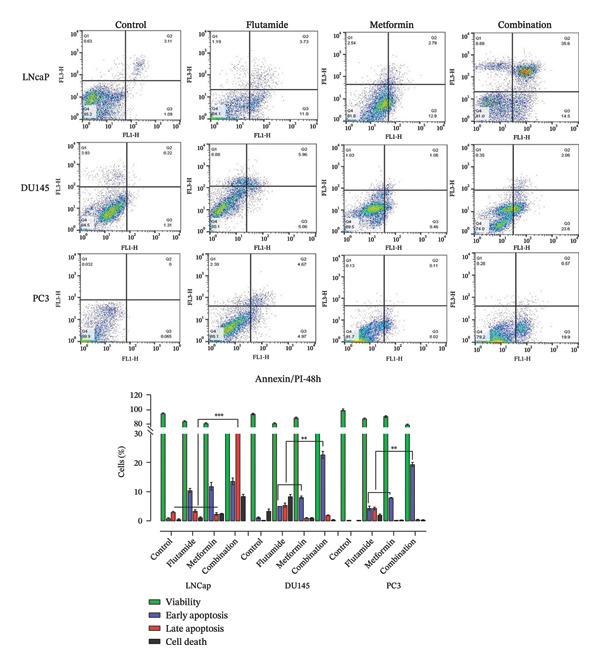
Evaluation of the apoptotic response in prostate cancer cell lines (LNCaP, DU145, and PC3) was performed using flow cytometry. The combination therapy of metformin and flutamide resulted in a higher number of apoptotic cells compared to other experimental groups. Statistical significance was observed at ^∗^ *p* < 0.05, ^∗∗^ *p* < 0.01, ^∗∗∗^ *p* < 0.001, and ^∗∗∗∗^ *p* < 0.0001 relative to the control group.

**FIGURE 6:**
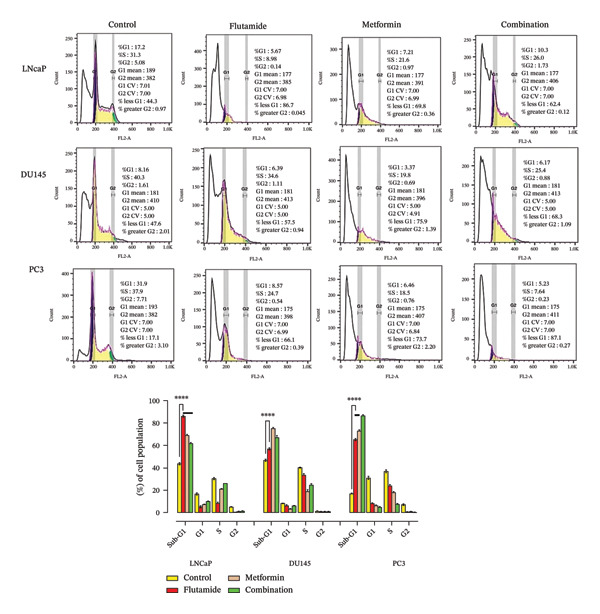
Cell cycle assay. Cell cycle analysis of prostate cancer cells (LNCaP, DU145, and PC3) was performed using flow cytometry. Using a PI staining kit, the analysis showed an increased sub‐G1 population in the treated groups compared to the corresponding controls, indicating enhanced apoptosis.

Assessment of the apoptotic response in PCa cell lines (LNCaP, DU145, and PC3) showed that the combination therapy of metformin and flutamide resulted in a higher number of apoptotic cells (Figure 5). Cell cycle analysis of LNCaP, DU145, and PC3 cells following treatment revealed distinct responses among the cell lines. Flutamide treatment in LNCaP cells, metformin treatment in DU145 cells, and metformin–flutamide treatment in PC3 cells led to a greater accumulation of cells in the sub‐G1 phase. Notably, in all treated groups, cells in the sub‐G1 stage were increased compared to their respective controls. Subsequently, drugs induced apoptosis, at least in part, by promoting G0/G1 cell cycle arrest in PCa cells (Figure 6). The cells in phase sub‐G1, respectively, control, flutamide, metformin, and combination in cell lines LNCaP, DU145, and PC3 are as follows: in the LNCaP cell line: 44.3, 86.7, 69.8, and 62.4, in the DU145 cell line: 47.6, 57.5, 75.9, and 68.3, and finally, in the PC3 cell line: 17.1, 66.1, 73.7, and 87.1. The highest sub‐G1 in the LNCaP cell line was related to the flutamide‐treated group, in the DU145 cell line to the metformin‐treated group, and in the PC3 cell line to the combination‐treated group.

### 2.6. Effect of Metformin and Flutamide and Their Combination on Gene Expression Levels in LNCaP, DU145, and PC3 Cells

Expression analyses of apoptosis‐related genes (*BAX/BCL2*), EMT‐related genes (*E-cadherin* and *Snail*), angiogenesis‐related genes (*HIF1α* and *VEGFC*), and the PCa biomarker (*KLK3*) were performed in LNCaP, DU145, and PC3 cells following treatments with metformin, flutamide, or metformin–flutamide combination.

**FIGURE 7:**
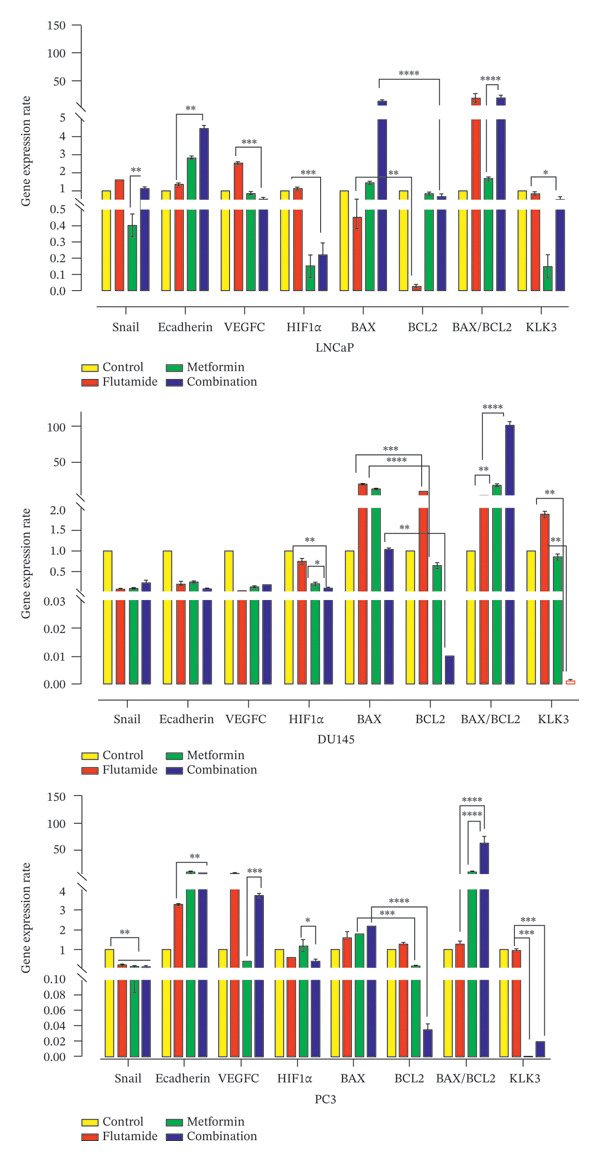
Real‐time PCR. The fold change results of prostate cancer cells (LNCaP, DU145, and PC3). Apoptosis‐related genes (*BAX/BCL2*), EMT‐related genes (*E-cadherin* and *snail*), angiogenesis‐related genes (*HIF1α* and *VEGFC*), and prostate cancer biomarkers (*KLK3*) were evaluated following treatment with the drugs at their IC50 concentrations. Favorable changes were seen in *KLK3*, *HIF1α*, and *E-cadherin* expression in LNCaP cells; in *HIF1α* and *BAX*/*BCL2* expression in DU145 cells; and in *VEGFC*, *HIF1α*, *BAX*/*BCL2*, *E-cadherin*, and *Snail* expression in PC3 cells. Statistical significance is indicated as follows: ^∗^ *p* < 0.05, ^∗∗^ *p* < 0.01, ^∗∗∗^ *p* < 0.001, and ^∗∗∗∗^ *p* < 0.0001 compared to the control group.

As demonstrated in Figure 7, treatment‐induced changes in gene expression varied across LNCaP, DU145, and PC3 cells. Specifically, the gene expression of *E-cadherin*, *Snail*, and *KLK3* genes was changed favorably in LNCaP cells. In contrast, DU145 cells showed more notable changes in apoptosis‐ and angiogenesis‐related genes, while in PC3 cells, the most significant effects were observed in the expression of apoptosis‐related genes and *KLK3*. It should be mentioned that comparing gene expression levels with their assigned controls showed statistically significant changes in all comparisons (*p* values < 0.05). In all three cell lines, the expression of genes related to apoptosis reflected a strong apoptotic response to both metformin and flutamide monotherapies, with further enhancement upon combination treatment. The combination of metformin and flutamide significantly upregulated the expression of *BAX/BCL2* and *E-cadherin* in all three cell lines (*p* < 0.001). Additionally, it significantly downregulated the expression of *KLK3, HIF1α*, *VEGFC*, and *SNAIL* genes in PC3 and LNCaP cells (*p* < 0.01).

### 2.7. Side Effects of Metformin and Flutamide on Liver Function

**FIGURE 8:**
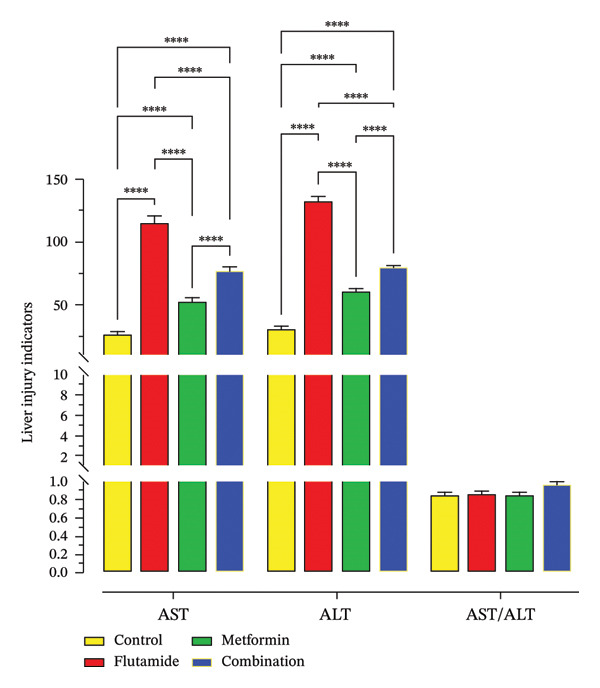
Hepatotoxicity assessment. Alterations in liver injury indicators. The average of liver function tests is shown in this diagram. ALT and AST are measured in international units per liter (U/L). ALT and AST levels were assessed using a colorimetric method. Metformin alone induces less liver damage compared to flutamide alone, and the combination of metformin and flutamide appeared to mitigate the hepatotoxic effects of flutamide on HepG2 cells. Statistical significance was distinct at *p* < 0.05, ^∗∗^ *p* < 0.01, ^∗∗∗^ *p* < 0.001, and ^∗∗∗∗^ *p* < 0.0001 compared to the control group.

Figure 8 shows liver function test results following treatment with metformin IC50, flutamide IC50, and their combination. Our investigation indicated that combining metformin with flutamide may reduce liver injury. The average results from three independent liver function tests revealed decreased ALT and AST levels in the metformin–flutamide combination therapy compared to flutamide alone. However, hepatotoxicity remained notable when compared to the control group, and finally, AST/ALT levels were not statistically significant in all groups.

## 3. Discussion

Although metformin–flutamide combination therapy has previously been evaluated in polycystic ovary syndrome for improving patients’ metabolic status [12], our study is the first to investigate its effects on PCa cells.

Altogether, our result demonstrated that combining metformin with flutamide enhances anticancerous effects in LNCaP, DU145, and PC3 cell lines. This synergistic interaction may not only improve patient prognosis but also reduce liver injury.

The combination treatment induced the highest levels of early apoptosis, with minimal late apoptosis and necrosis in PC3 and DU145 cells, respectively. In contrast, LNCaP cells exhibited both the highest rates of apoptosis and necrosis. Furthermore, co‐administration with metformin allowed for a reduction in flutamide dosage while maintaining its efficacy and mitigating its adverse effects.

Metformin, an antihyperglycemic agent, primarily disrupts the mitochondrial respiratory chain by inhibiting complex I. This inhibition reduces ATP production while elevating AMP levels, thereby increasing the AMP/ATP ratio. As a result, AMPK, a key energy‐sensing enzyme, is directly activated, influencing various metabolic pathways. Additionally, metformin exerts AMPK‐independent effects, such as suppressing fructose‐1,6‐bisphosphatase, an enzyme involved in glucose synthesis [13].

The mechanism of action of flutamide, a nonsteroidal antiandrogen, has been explored in studies involving the accessory sex tissues of male rats. Flutamide effectively inhibits the activity of both endogenous and externally administered testosterone, suppresses testosterone‐induced prostatic DNA synthesis, and inhibits the nuclear uptake of androgens in the prostatic tissue [14].

Importantly, Yasui et al. declared PCa patients can benefit from changing their antiandrogen regimen, for example, switching from bicalutamide to flutamide, upon tumor relapses and that treatment outcomes can be predicted using indicators such as PSA levels. This study supports the continued clinical relevance of flutamide as a valuable therapeutic choice for both health care providers and patients [15].

Given that flutamide has been shown to reduce PSA levels, and considering the prognostic value of PSA in monitoring tumor recurrence [16, 17], we investigated the expression of the KLK3 gene (prostate‐specific antigen, PSA) following treatment with flutamide, metformin, and the combination of flutamide and metformin. Our results demonstrated that the combination of metformin and flutamide significantly downregulated KLK3 expression in PCa cell lines.

On the other hand, several studies reported antitumor properties of metformin in PCa, mediated through various mechanisms of action [18–20]. Alghandour et al. showed that metformin, as a safe and inexpensive drug, combined with ADT, ameliorates the PCa footprint, mostly in locally advanced or metastatic types [21]. Additionally, a real‐world study, published in 2023, involving over 100,000 individuals, also reports metformin usage can lessen PCa‐attributed deaths [22].

The STAMPEDE trial is a randomized controlled study that recruits patients with locally advanced or metastatic PCa undergoing first‐line hormone therapy. This multiarm, multistage platform evaluates the efficacy of various treatment combinations alongside standard ADT [23]. Recently, a manuscript from the metformin arm of the trial was published. In this study involving approximately 1900 men with metastatic hormone‐sensitive PCa, the addition of metformin to standard treatment did not improve overall survival. However, it was associated with a significant reduction in the metabolic side effects of ADT [24]. It is worth mentioning that the hormonal therapy in this study included orchidectomy, luteinizing hormone‐releasing hormone (LHRH)‐based treatments, or bicalutamide [25].

Moreover, several studies have evaluated the association between metformin’s protective potential and the risk of PCa development; however, the findings remain controversial [26]. Exploring the NIH clinical trials database demonstrates more than 300 trials assessing metformin’s impact in different cancers. Among them, about 30 trials evaluate the metformin and PCa association [27].

Interestingly, Kong et al. investigated the effects of metformin, bicalutamide, and their combination on PC3 and LNCaP cells, reporting that both single and combined treatments significantly reduced clonogenicity. This effect was more pronounced in the combination group and particularly evident in AR‐positive cells. Consistent with their findings, our results show that the combination of metformin with even the first‐generation antiandrogen flutamide produced similar effects, supporting the potential of such combinations in targeting AR‐positive and AR‐negative PCa cells [28].

Additionally, our investigation revealed that metformin addition to flutamide resulted in increased apoptosis, while the level of necrosis remained negligible. Yang et al. also reported combining metformin with ADT (culturing in charcoal‐stripped serum or bicalutamide treatment) induced apoptosis in PCa cells. The study indicated an additive inhibitory impact of the combination in both in vivo and in vitro analyses [8].

Another research study by Wang et al. analyzed metformin and bicalutamide treatment in androgen‐dependent and independent cell lines, LNCaP and 22RV1, respectively [29].

The 22Rv1 cell line was established from the CWR22 PCa xenograft following an extended period of androgen‐deprivation therapy. This cell line is notable for the presence of the androgen receptor [2], which includes both mutated and alternatively spliced variants such as AR‐V7, exhibiting constitutive transcriptional activity that is independent of androgens. Consequently, the 22Rv1 line is not entirely androgen‐independent, as it continues to express AR and shows partial responsiveness to androgens. Nonetheless, it demonstrates characteristics of partial androgen independence, rendering it a well‐recognized model for castration‐resistant prostate cancer (CRPC) [30]. Wang et al. reported that metformin induced apoptosis and diminished cell viability in both PCa cell lines. Treating LNCaP cells with the mentioned combination represented also synergistic effects. Besides, Wang et al. declared metformin causes G1/S cell cycle arrest [29]. In contrast, our results showed that metformin, both alone and in combination with flutamide, induced cell cycle arrest at the G0/G1 phase in the LNCaP cell line.

Chen et al. investigated the effect of metformin on DU145 and PC3 cells and reported that the drug enhances antiproliferation features and triggers G0/G1 cell cycle arrest. Their study also showed that metformin’s antitumor effects in PCa are mediated through activation of autophagy signaling pathways [31]. In accordance with the recent statement, we found combined metformin and flutamide treatment led to cell cycle arrest at the G0/G1 phase in PCa cells.

In addition to studies investigating the combination of metformin with bicalutamide, the co‐administration of metformin and docetaxel has also been examined in PCa cells. Kang et al. reported that exposure to a metformin–docetaxel combination significantly decreased cell viability in PC3 cells, though it did not affect cancer cell migration [32]. Notably, metformin alone was also capable of reducing the viability of PCa cell lines [33]. In line with findings of this study, the combination of metformin with a reduced dose of flutamide demonstrated the most pronounced effect on the prostate metastatic cell line (PC3), enhancing apoptosis while reducing cell viability and migratory capacity.

Resistance of PCa cells to antiandrogens could be due to their reprogramming and acquiring stem cell characteristics. Biological studies have shown that metformin can overcome enzalutamide resistance [34]. Goncharov et al. and Chaves et al. investigated the effects of metformin, enzalutamide, and their combination on PCa cells, demonstrating that metformin can restore enzalutamide sensitivity by inhibiting epithelial–mesenchymal transition (EMT) [11, 35].

Chen et al. revealed that metformin, alone or in combination with castration, reduces EMT by upregulating the epithelial *E-cadherin* level. Consequently, the treatment hinders invasion and migration of PCa cells [36]. In accordance with this finding, our result demonstrated that metformin–flutamide treatment suppresses *EMT* in LNCaP cells, while this favorable outcome was recognized in PC3 cells more significantly.

As mentioned earlier, flutamide’s hepatotoxicity is a known disadvantage of the drug [37, 38]. Recently published evidence suggested that metformin may diminish liver injury in vivo [39]. Our analysis similarly demonstrated that metformin addition to flutamide can attenuate liver damage.

For the first time, we showed that metformin–flutamide combination treatment is effective against PCa cells. According to the obtained results, this combination is more potent in metastatic cancer cells (PC3), including increased apoptosis, reduced necrosis, cell cycle arrest at the G0/G1 phase, and decreased expression of EMT marker key factors in tumor progression. These findings suggest that this novel combination therapy may offer a safer and more effective treatment option. Additionally, it was shown for the first time that metformin co‐administration allows for flutamide dose reduction, potentially minimizing flutamide‐related liver complications.

## 4. Limitations and Future Directions

This study was conducted in vitro using only three cell lines, which may limit generalizability due to cell line‐specific characteristics and the absence of a tumor microenvironment and systemic influences. Drug concentrations used may not directly correspond to clinically achievable doses, and no in vivo validation was performed. Future studies should include animal models to assess pharmacologic relevance, evaluate dose–response relationships in vivo, and ultimately progress to clinical trials to confirm translational potential.

## Funding

The authors received no specific funding for this work.

## Ethics Statement

This study was approved by the ethics committee of Tehran University of Medical Sciences, Tehran, Iran (IR.TUMS.MEDICINE.REC.1401.495).

## Conflicts of Interest

The authors declare no conflicts of interest.

## Data Availability

Information, data, and photos will be provided upon request.
